# Lymph node metastasis risk factors in T2 colorectal cancer

**DOI:** 10.1002/deo2.70040

**Published:** 2024-11-29

**Authors:** Yuriko Morita, Shin‐ei Kudo, Yuki Takashina, Katsuro Ichimasa, Yuta Kouyama, Shigenori Semba, Kenichi Mochizuki, Osamu Shiina, Shun Kato, Takanori Kuroki, Shoji Shimada, Kenta Nakahara, Yusuke Takehara, Shunpei Mukai, Noriyuki Ogata, Takemasa Hayashi, Kunihiko Wakamura, Hideyuki Miyachi, Naruhiko Sawada, Tetsuo Nemoto, Toshiyuki Baba, Masashi Misawa

**Affiliations:** ^1^ Digestive Disease Center Showa University Northern Yokohama Hospital Kanagawa Japan; ^2^ Department of Medicine National University of Singapore Singapore Singapore; ^3^ Department of Gastroenterology and Endoscopy, Kochi Medical School Kochi University Kochi Japan; ^4^ Pathology Department Showa University Northern Yokohama Hospital Kanagawa Japan

**Keywords:** clinical, colorectal neoplasms, lymph nodes, neoplasm metastasis, pathology, risk factors

## Abstract

**Objectives:**

This study evaluates risk factors for lymph node metastasis (LNM) in T2 colorectal cancer to refine patient selection for endoscopic resection.

**Methods:**

We reviewed records from consecutive patients who had undergone curative surgical resection of T2 colorectal cancer at our institution in Japan between April 2001 and December 2021. Data on conventional clinicopathologic variables were retrieved from the pathology reports at the time of surgery. The clinicopathological features included patient age, sex, tumor diameter, morphology, tumor location, lymphatic invasion, vascular invasion, tumor differentiation, carcinoembryonic antigen and carbohydrate antigen 19‐9 levels, number of lymph node dissections, presence of adenoma component, and LNM.

**Results:**

Among the patients (338 men, 320 women), 170 (25.8%) exhibited LNM. Multivariate logistic regression identified three independent risk factors for LNM: lymphatic invasion (odds ratio [OR], 32.6; 95% confidence interval [CI], 17.3–61.4; *p* < 0.0001), female sex (OR, 1.70; 95% CI, 1.10–2.62; *p* = 0.02), and elevated carcinoembryonic antigen levels (OR, 2.56; 95% CI, 1.10–5.96; *p* = 0.03).

**Conclusions:**

Lymphatic invasion, female sex, and high carcinoembryonic antigen levels significantly increase the risk of LNM in T2 colorectal cancer.

## INTRODUCTION

Advances in endoscopic techniques, including colorectal polypectomy, endoscopic mucosal resection, and endoscopic submucosal dissection, coupled with the use of magnifying endoscopes for pit pattern diagnosis, have enhanced the diagnostic and therapeutic management of T1 colorectal cancer (CRC). Given that approximately 10% of T1 CRC cases develop lymph node metastasis (LNM), the Japanese Society for Cancer of the Colon and Rectum guidelines advocate for additional surgical resection with lymph node dissection for high‐risk LNM patients following endoscopic resection. Identified risk factors include depth of submucosal invasion, lymphovascular invasion, poor differentiation, and tumor budding.^1^, ^2^

Conversely, T2 CRC, characterized by muscularis propria invasion, presents with LNM in about 25% of cases, with surgery and lymph node dissection being the standard of care.^1^ However, emerging endoscopic techniques such as endoscopic full‐thickness resection (eFTR), endoscopic intermuscular dissection (EID), and per‐anal endoscopic myectomy (PAEM) have the potential to shift the treatment paradigms.^3^, ^4^, ^5^ These new techniques offer a “resect and analysis” approach, in which T2 CRC is endoscopically resected, and the necessity for subsequent surgery is determined according to the histopathological evaluation of LNM risk.^6^, ^7^, ^8^ This approach has shown promise in case reports and could be particularly beneficial for elderly patients or those ineligible for surgery due to comorbid conditions.^9^, ^10^

Although some previous reports investigated risk factors of LNM in T2 CRC, most of them were limited to rectal or colon T2 cancer or small cases.^11^, ^12^, ^13^, ^14^ There are also no reports stratifying the risk of LNM combining these risk factors. This study aims to define the patients with very low risk of LNM to expand the number of cases in which endoscopic resection is a viable treatment option by discovering the important risk factors associated with LNM in T2 CRC.

## METHODS

### Eligibility criteria

This was a single‐center retrospective study. Data on patients resected surgically for pathology‐verified T2 CRC at Showa University Northern Yokohama Hospital in Japan from April 1, 2001, to December 31, 2021, were included in this study.

The following patients were ineligible for inclusion in the study: synchronous CRC; transanal endoscopic microsurgery; familial adenomatous polyposis, Lynch syndrome, or inflammatory bowel disease; preoperative chemotherapy or radiotherapy; and missing data. The following clinicopathological characteristics were analyzed: patient age, sex, carcinoembryonic antigen (CEA; ng/mL) and carbohydrate antigen 19‐9 (CA19‐9; U/mL) serum levels, tumor size, location, differentiation status, lymphatic invasion, vascular invasion, presence of adenoma component, number of lymph nodes dissected, and LNM at the time of surgery.

### Surgical procedures

This study classified the N stage and scope of lymph node dissection according to the Japanese Classification of Colorectal, Appendiceal, and Anal Carcinoma.^15^ N1 is defined as metastasis to epicolic and paracolic lymph nodes located within 5 cm of the tumor, N2 as metastasis to pericolic lymph nodes situated 5–10 cm away from the tumor and intermediate lymph nodes along the primary feeding artery, and N3 as main lymph nodes at the root of the primary feeding artery. All of these are defined as regional lymph nodes. D1 lymph node dissection is defined as the removal of pericolic nodes located within 5 cm of the tumor, D2 dissection as the removal of pericolic nodes located within 10 cm of the tumor and intermediate nodes, and D3 dissection as the removal of all regional nodes including main nodes. In our institution, D3 dissection was generally performed, with D2 dissection being performed for very elderly patients or those with high surgical risk. Lateral lymph node dissection for rectal cancer located below the peritoneal reflection was not indicated except if lateral LNM is not detected by a preoperative or intraoperative diagnosis, according to the guidelines.^1^

### Histological examination

The resected specimens were fixed with 10% buffered formalin, and the tumor‐containing tissue samples were sliced into 4–5 mm sections at the part with the deepest tumor invasion. Histopathological diagnoses were established by hematoxylin and eosin staining following the procedures described in the Japanese Classification.^15^ All specimens were diagnosed using the 2019 World Health Organization Classification of Tumors and the current Japanese Society for Cancer of the Colon and Rectum guidelines.^1^, ^16^

### Statistical analysis

Normally distributed continuous variables were reported as the mean ± standard deviation, whereas non‐normally distributed variables were reported as the median (interquartile range). Normally distributed continuous variables were compared using Student's *t*‐tests, whereas non‐normally distributed variables were analyzed using the Wilcoxon rank‐sum test. Dichotomous variables were compared using chi‐squared or Fisher's exact tests, as appropriate. Multivariate logistic regression analysis regarding LNM was subsequently performed to calculate the odds ratio (OR) and 95% confidence interval (CI). Variables with a *p*‐value of less than 0.1 in the univariate analysis were selected for inclusion in the multivariate analysis. All statistical analyses were performed using JMP Pro version 16.0.0 (SAS Institute Inc., Cary, NC, USA). All *p*‐values were two‐sided, and *p* < 0.05 was considered statistically significant.

## RESULTS

### Study subjects

**FIGURE 1:**
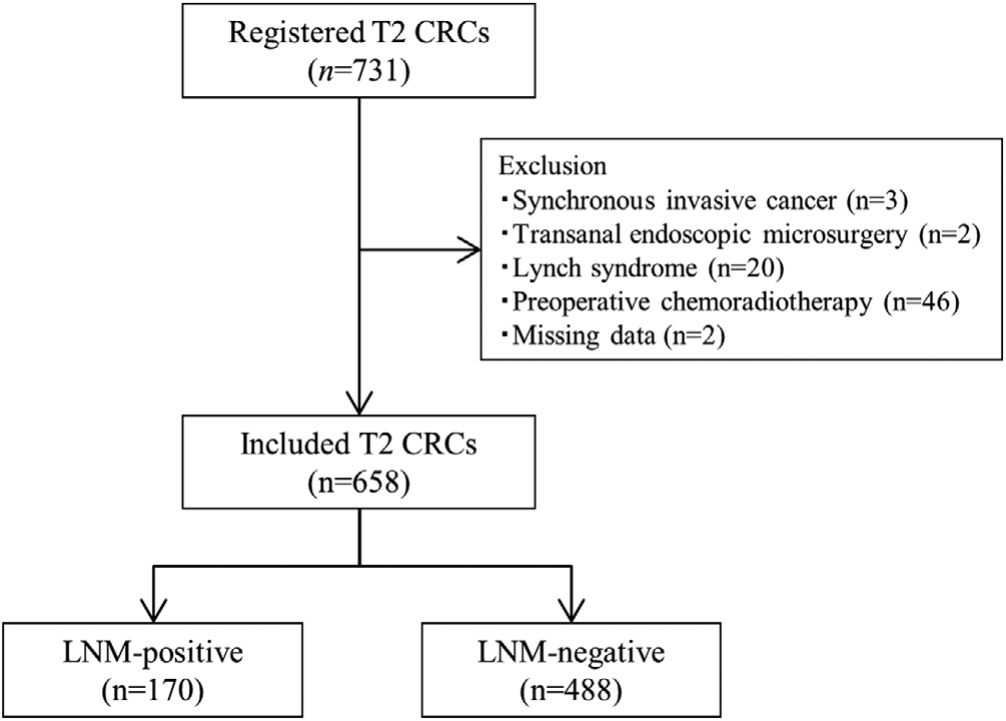
Patients' flow chart.

Figure 1 shows the study flow chart. Altogether, 731 patients with pT2 CRC were treated during the study period. Of these, 73 patients were excluded (synchronous invasive cancer; *n* = 3, transanal endoscopic microsurgery; *n* = 2, Lynch syndrome; *n* = 20, preoperative chemoradiotherapy; *n* = 46, and missing data; *n* = 2), and 658 were eligible and included in the analyses. The LNM rate was 25.8% (170/658; 95% CI, 22.5–29.4). Among these cases, 21.9% (144/658; 18.8–25.2), 2.7% (18/658; 1.6–4.3), and 1.2% (8/658; 0.5–2.4) were accompanied by N1, N2 and N3 metastasis, respectively. The mean number of retrieved lymph nodes per patient was 21, and the median was 19. The clinical characteristics of the patients are shown in Table 1. The median age of the patients was 69 years; 338 (51.2%) were male, with a median CEA level of 1.8 (1.1–2.7) and a CA19‐9 level of 10.5 (6.2–17.7). The median tumor diameter was 28 mm (21–35), and 47.2% were located on the left side of the colon. Lymphatic and vascular invasion positivity presented in 297 (45.1%) and 388 (59.0%) cases, respectively.

**TABLE 1:** Characteristics of patients at baseline (*n* = 658).

Age (years)	69 (59–76)
Sex (male/female)	338 (51.4)/320 (48.6)
CEA	1.8 (1.1–2.7)
CA19‐9	10.5 (6.2–17.7)
Tumor size (mm)	28 (21–35)
Location (left/right)	472 (71.7)/186 (28.3)
Histological differentiation Predominant (por/muc/sig)	19 (2.9)
Histological differentiation Highest‐grade (por/muc/sig)	126 (19.1)
Lymphatic invasion (+)	297 (45.1)
Vascular invasion (+)	388 (59.0)
Adenomatous component (+)	22 (3.3)
Number of lymph nodes dissected	20 (13–28)
LNM‐positive (+)	170 (25.8)

*Note*: Results are expressed as median (interquartile range) or number of patients (%), as appropriate.

Abbreviations: CA19‐9, carbohydrate antigen 19‐9; CEA, carcinoembryonic antigen; muc, mucinous carcinoma; por, poorly differentiated adenocarcinoma; sig, signet‐ring cell carcinoma.

### Correlation between the clinicopathological factors and LNM

Table 2 illustrates the association between various clinicopathological factors and the presence of LNM in patients with T2 CRC. Out of 658 cases, 170 (25.8%) exhibited LNM. Analyses of clinicopathological data identified significant associations: females showed a higher incidence of LNM, with 96 out of 170 cases (56.5%) positive for LNM (*p* = 0.02). Lymphatic invasion, present in 158 of 170 cases (92.9%), was associated with LNM (*p* < 0.0001). Similarly, vascular invasion was present in 124 out of 170 cases (72.9%) with LNM, which also showed a correlation (*p* < 0.0001). Other factors analyzed did not show a statistically significant association with LNM (Table 2).

**TABLE 2:** Relationship between clinicopathological factors and lymph node metastasis status in T2 colorectal cancer (*n* = 658).

	LNM positive (*n* = 170)	LNM negative (*n* = 488)	*p*‐value
Age (years)	68 (58–75)	69 (60–76)	0.09
Sex			
Male	74 (43.5)	264 (54.1)	0.02
Female	96 (56.5)	224 (45.9)	
CEA			
<5.0 ng/mL	152 (89.4)	458 (93.9)	0.06
≥5.0 ng/mL	18 (10.6)	30 (6.1)	
CA19‐9			
<37.0 U/mL	153 (90.0)	457 (93.6)	0.12
≧37.0 U/mL	17 (10.0)	31 (6.4)	
Tumor size (mm)			
<20 mm	32 (18.8)	87 (17.8)	0.82
≧20 mm	138 (81.2)	401 (82.2)	
Location			
Right	39 (22.9)	147 (30.1)	0.08
Left	131 (77.1)	341 (69.9)	
Histological differentiation (predominant)			
Well/mode	164 (96.5)	475 (97.3)	0.60
por/muc/sig	6 (3.5)	13 (2.7)	
Histological differentiation (highest‐grade)			
Well/mode	131 (77.1)	401 (82.2)	0.17
por/muc/sig	39 (22.9)	87 (17.8)	
Lymphatic invasion			
Positive	158 (92.9)	139 (28.5)	<0.0001
Negative	12(7.1)	349 (71.5)	
Vascular invasion			
Positive	124 (72.9)	264 (54.1)	<0.0001
Negative	46 (27.1)	224 (45.9)	
Adenomatous component			
Present	8 (4.7)	14 (2.9)	0.25
Absent	162 (95.3)	474 (97.1)	
Number of lymph nodes dissected	20 (13–29)	19 (12–28)	0.30

*Note*: Results are expressed as median (interquartile range) or number of patients (%), as appropriate.

Abbreviations: CA19‐9, carbohydrate antigen 19‐9; CEA, carcinoembryonic antigen; LNM, lymph node metastasis; mod, moderately differentiated adenocarcinoma; muc, mucinous carcinoma; por, poorly differentiated adenocarcinoma; sig, signet‐ring cell carcinoma; well, well‐differentiated adenocarcinoma.

### Multivariate analysis of risk factors for LNM and risk stratification

Table 3 delineates the associations between clinicopathologic factors and LNM, as determined by multivariate logistic regression analysis. These analyses identified several factors significantly associated with an increased risk of LNM. Lymphatic invasion emerged as the most potent risk factor, with an OR of 32.6 (95% CI, 17.3–61.4; *p* < 0.0001). Additionally, female sex was associated with a moderately increased risk (OR, 1.70; 95% CI,1.10–2.62; *p* = 0.02). Elevated CEA levels (>5.0 ng/mL) also significantly correlated with LNM, with an OR of 2.56 (95% CI, 1.10–5.96; *p* = 0.03). The risk of LNM was stratified according to the number and combination of risk factors, as shown in Table 4. Cases without lymphatic invasion accounted for 54.9% (361/658; 95% CI, 51.0–58.7), with an LNM rate of 3.3% (12/361; 95% CI, 1.7–5.7). Conversely, cases with lymphatic invasion had an LNM rate of 53.2% (158/297; 95% CI, 47.3–59.0).

**TABLE 3:** Multivariate analysis of risk factors for lymph node metastasis in T2 colorectal cancer.

Variables	OR	95% CI	*p*‐value
Age (years)	0.54	0.16–1.85	0.32
Sex (female)	1.70	1.10–2.62	0.02
CEA (≥5.0 ng/mL)	2.56	1.10–5.96	0.03
Location (left)	1.07	0.64–1.79	0.80
Lymphatic invasion (+)	32.6	17.3–61.4	<0.0001
Vascular invasion (+)	1.39	0.86–2.23	0.17

Abbreviations: CEA, carcinoembryonic antigen; 95% CI, 95% confidence interval; OR, odds ratio.

**TABLE 4:** Risk stratification of lymph node metastasis in T2 colorectal cancer.

Number of risk factors	Patients (*n* = 658)	LNM‐positive (*n* = 170)	Rate of LNM (%) (95% CI)	Rate of LNM (%) (95% CI)
None	175	4	2.3 (0.6–5.7)	3.3 (1.7–5.7)
1‐2 (female or/and CEA)	186	8	4.3 (1.9–8.3)	
1 (Ly)	141	63	44.7 (36.3–53.3)	53.2 (47.3–59.0)
2 (Ly and female)	134	77	57.5 (48.6–66.0)	
2 (Ly and CEA)	9	7	77.8 (40.0–97.2)	
3 (Ly, female, and CEA)	13	11	84.6 (54.6–98.1)	

Abbreviations: CEA, carcinoembryonic antigen; LNM, lymph node metastasis; Ly, lymphatic invasion; 95% CI, 95% confidence interval.

## DISCUSSION

This study evaluated the clinicopathologic characteristics of T2 CRC to determine risk stratification and identify cases potentially treatable solely through endoscopic methods. The multivariate analysis identified lymphatic invasion, female sex, and elevated serum CEA (≥5.0 ng/mL) as statistically significant factors for LNM in T2 CRC at the time of surgery.

The development of endoscopic techniques such as eFTR, EID, and PAEM has expanded the range of lesions treatable endoscopically. Current indications for eFTR and EID include the primary treatment of optically diagnosed T1 CRC, non‐lifted lesions, severe fibrosis, and secondary treatment after incomplete endoscopic resection. Recent reports have documented the successful endoscopic resection of T2 CRC with negative margins, particularly in patients who are poor surgical candidates.^9^ Given the overall postoperative mortality rate for CRC surgery of 1.3%–8.0%, with specific rates increasing with patient age, endoscopic treatment may offer a viable alternative for low‐risk LNM patients with T2 CRC.^17^ In this study, the LNM rate in patients without lymphatic invasion was 3.3% (95% CI, 1.7–5.7), a rate that may be considered acceptable when compared to the postoperative mortality rate. In contrast, the LNM rate in patients with lymphatic invasion—the factor most strongly associated with LNM—exceeded 53.2% (95% CI, 47.3‐59.0), making surgical intervention highly recommended. Although the present study is limited by a small sample size, further stratification of LNM risk may be possible by incorporating female sex and CEA levels, which are independent risk factors for LNM in this study as the sample size increases. However, there is no evidence regarding overall or disease‐free survival rates. In T1 CRC, the low‐risk group for LNM has been well‐defined, with oncologic safety as the highest priority, and an acceptable long‐term prognosis has been documented. As new treatment strategies for T2 CRC are introduced, it is imperative to establish stricter criteria. Drawing from previous findings, potential endoscopic treatment indications for T2 CRC have been suggested: (1) eFTR for lesions smaller than 20 mm, regardless of location, or (2) EID/PAEM for rectal tumors, irrespective of size.^7^ Preoperative diagnosis of the depth of invasion will be essential for these strategies to distinguish T2 cancer from T1/T3 cancer. There is no systematic pit pattern classification or narrow band imaging classification for diagnosing the depth of invasion of T2 CRC, but endoscopic ultrasound may be effective.^18^ Previous reports have shown that the diagnostic accuracy of the T stage was 94% with no under‐staging.^19^

Previous studies have consistently reported lymphovascular invasion as a reliable risk factor for T1 and T2 CRC.^20^, ^21^, ^22^ This study supports those findings and highlights additional common risk factors, such as female sex. This result agrees with findings from previous reports of T1 CRC, which identified female sex as a risk factor for LNM.^23^, ^24^ Although the underlying reasons for this trend are unclear, it may be associated with hormone levels, particularly the role of estrogen and its receptors.^25^, ^26^ This hypothesis suggests that hormonal differences could affect tumor behavior and metastatic potential and warrants further investigation to understand the mechanisms and potential implications for treatment strategies. Despite the known correlation of CEA levels with prognosis in advanced CRC, we show a correlation with LNM, specifically in T2 CRC.^21^, ^27^ In the future, the use of circulating tumor DNA is expected to enable the development of predictive models that are more accurate than those based on non‐invasive methods, leading to a more precise delineation of the treatment indications.^28^

This study has some limitations. First, it is a single‐center, retrospective analysis. Second, while the degree of invasion has been recognized as a risk factor in T1 CRC, it was not assessed in this study since it was not assessed routinely in T2 CRC. In addition, tumor budding and perineural invasion were not included in this study because they had been identified as prognostic factors only since 2013 in the Japanese guidelines.^15^ This may result in potential risk factors for LNM being overlooked. Third, the degree of vascular invasion‐positivity was subdivided from three to four levels in 2018 in the Japanese guidelines. Therefore, we stratified the degree of vascular invasion into two categories, positive or negative, for analysis and lymphatic invasion.

In summary, lymphatic invasion, female sex, and elevated CEA levels were identified as independent risk factors for LNM in T2 CRC. Combining these risk factors may enable stratification of LNM risk.

## CONFLICT OF INTEREST STATEMENT

None.

## ETHICS STATEMENT

This study protocol was approved by the institutional review board of Showa University (approval No.21‐106‐B).

## PATIENT CONSENT STATEMENT

Written informed consent was obtained from all patients before treatment.

## CLINICAL TRIAL REGISTRATION

This study was registered in the University Hospital Medical Network Clinical Trials Registry (UMIN000046992).

## ANIMAL STUDIES

N/A.

## References

[1] Hashiguchi Y , Muro K , Saito Y *et al.* Japanese Society for Cancer of the Colon and Rectum (JSCCR) guidelines 2019 for the treatment of colorectal cancer. Int J Clin Oncol 2020; 25: 1–42. 31203527 10.1007/s10147-019-01485-z PMC6946738

[2] Ichimasa K , Kudo SE , Miyachi H *et al.* Current problems and perspectives of pathological risk factors for lymph node metastasis in T1 colorectal cancer: Systematic review. Dig Endosc 2022; 34: 901–912. 34942683 10.1111/den.14220

[3] Zwager LW , Bastiaansen BAJ , van der Spek BW *et al.* Endoscopic full‐thickness resection of T1 colorectal cancers: A retrospective analysis from a multicenter Dutch eFTR registry. Endoscopy 2022; 54: 475–485. 34488228 10.1055/a-1637-9051

[4] Moons LMG , Bastiaansen BAJ , Richir MC *et al.* Endoscopic intermuscular dissection for deep submucosal invasive cancer in the rectum: A new endoscopic approach. Endoscopy 2022; 54: 993–998. 35073588 10.1055/a-1748-8573

[5] Toyonaga T , Ohara Y , Baba S *et al.* Peranal endoscopic myectomy (PAEM) for rectal lesions with severe fibrosis and exhibiting the muscle‐retracting sign. Endoscopy 2018; 50: 813–817. 29883977 10.1055/a-0602-3905

[6] Ichimasa K , Nakahara K , Kudo SE *et al.* Novel “resect and analysis” approach for T2 colorectal cancer with use of artificial intelligence. Gastrointest Endosc 2022; 96: 665–672.e1. 35500659 10.1016/j.gie.2022.04.1305

[7] Ichimasa K , Kudo SE , Tan KK , Lee JWJ , Yeoh KG . Challenges in implementing endoscopic resection for T2 colorectal cancer. Gut Liver 2024; 18: 218–221. 37842729 10.5009/gnl230125 PMC10938148

[8] Ichimasa K , Foppa C , Kudo SE *et al.* Artificial intelligence to predict the risk of lymph node metastasis in T2 colorectal cancer. Ann Surg 2024; 280: 850–857. 39077765 10.1097/SLA.0000000000006469

[9] Wilson N , Abdallah M , Bilal M . Hybrid endoscopic submucosal dissection and endoscopic full‐thickness resection for complete resection of a T2 colorectal adenocarcinoma in a nonsurgical candidate. Gastrointest Endosc 2023; 98: 136–137. 36731580 10.1016/j.gie.2023.01.045

[10] Liu BR , Liu D , Ullah S , Zhao L , Zheng Q , Ullah R . Endoscopic full‐thickness resection and endoscopic lymphadenectomy for advanced colonic cancer in an inoperable patient: First human clinical experience. Gastrointest Endosc 2020; 91: 451–452. 31520589 10.1016/j.gie.2019.09.005

[11] Ushigome H , Ohue M , Kitamura M *et al.* Evaluation of risk factors for lymph node metastasis in T2 lower rectal cancer to perform chemoradiotherapy after local resection. Mol Clin Oncol 2020; 12: 390–394. 32190324 10.3892/mco.2020.1993 PMC7057958

[12] Tong LL , Gao P , Wang ZN *et al.* Is pT2 subclassification feasible to predict patient outcome in colorectal cancer? Ann Surg Oncol 2011; 18: 1389–1396. 21107740 10.1245/s10434-010-1440-2

[13] Komori K , Kanemitsu Y , Ishiguro S , Shimizu Y , Sano T , Kato T . Analysis of lymph node metastatic pattern according to the depth of in‐growth in the muscularis propria in T2 rectal cancer for lateral lymph node dissection. Dig Surg 2011; 28: 352–359. 22042151 10.1159/000332825

[14] Kotake K , Kobayashi H , Asano M , Ozawa H , Sugihara K . Influence of extent of lymph node dissection on survival for patients with pT2 colon cancer. Int J Colorectal Dis 2015; 30: 813–820. 25808013 10.1007/s00384-015-2194-x

[15] Japanese Society for Cancer of the Colon and Rectum . Japanese classification of colorectal, appendiceal, and anal carcinoma: The 3d English edition [Secondary Publication]. J Anus Rectum Colon 2019; 3: 175–195. 31768468 10.23922/jarc.2019-018 PMC6845287

[16] WHO Classification of Tumours Editorial Board . Digestive System Tumours, 5th edn, Lyon, France: IARC Publications, 2019.

[17] Jafari MD , Jafari F , Halabi WJ *et al.* Colorectal cancer resections in the aging US population: A trend toward decreasing rates and improved outcomes. JAMA Surg 2014; 149: 557–564. 24718844 10.1001/jamasurg.2013.4930

[18] Esaki M , Yamamura T , Nakamura M *et al.* Endoscopic ultrasound elastography as a novel diagnostic method for the assessment of hardness and depth of invasion in colorectal neoplasms. Digestion 2021; 102: 701–713. 33207360 10.1159/000511589

[19] Stergiou N , Haji‐Kermani N , Schneider C , Menke D , Kockerling F , Wehrmann T . Staging of colonic neoplasms by colonoscopic miniprobe ultrasonography. Int J Colorectal Dis 2003; 18: 445–449. 12783253 10.1007/s00384-003-0506-z

[20] Bosch SL , Teerenstra S , de Wilt JH , Cunningham C , Nagtegaal ID . Predicting lymph node metastasis in pT1 colorectal cancer: A systematic review of risk factors providing rationale for therapy decisions. Endoscopy 2013; 45: 827–834. 23884793 10.1055/s-0033-1344238

[21] Watanabe J , Ichimasa K , Kudo SE *et al.* Risk factors for lymph node metastasis in T2 colorectal cancer: A systematic review and meta‐analysis. Int J Clin Oncol 2024; 29: 921–931. 38709424 10.1007/s10147-024-02547-7

[22] Ichimasa K , Kudo SE , Miyachi H , Kouyama Y , Misawa M , Mori Y . Risk stratification of T1 colorectal cancer metastasis to lymph nodes: Current status and perspective. Gut Liver 2021; 15: 818–826. 33361548 10.5009/gnl20224 PMC8593512

[23] Kajiwara Y , Oka S , Tanaka S *et al.* Nomogram as a novel predictive tool for lymph node metastasis in T1 colorectal cancer treated with endoscopic resection: A nationwide, multicenter study. Gastrointest Endosc 2023; 97: 1119–1128.e5. 36669574 10.1016/j.gie.2023.01.022

[24] Ichimasa K , Kudo SE , Miyachi H *et al.* Patient gender as a factor associated with lymph node metastasis in T1 colorectal cancer: A systematic review and meta‐analysis. Mol Clin Oncol 2017; 6: 517–524. 28413659 10.3892/mco.2017.1172 PMC5374909

[25] Bosetti C , Bravi F , Negri E , La Vecchia C . Oral contraceptives and colorectal cancer risk: A systematic review and meta‐analysis. Hum Reprod Update 2009; 15: 489–498. 19414526 10.1093/humupd/dmp017

[26] Jassam N , Bell SM , Speirs V , Quirke P . Loss of expression of oestrogen receptor beta in colon cancer and its association with Dukes' staging. Oncol Rep 2005; 14: 17–21. 15944762

[27] Xiong X , Wang C , Cao J , Gao Z , Ye Y . Lymph node metastasis in T1‐2 colorectal cancer: A population‐based study. Int J Colorectal Dis 2023; 38: 94. 37055602 10.1007/s00384-023-04386-w

[28] Miyo M , Kato T , Nakamura Y *et al.* DENEB: Development of new criteria for curability after local excision of pathological T1 colorectal cancer using liquid biopsy. Cancer Sci 2022; 113: 1531–1534. 34839585 10.1111/cas.15226 PMC8990725

